# Sensorimotor stroke alters hippocampo-thalamic network activity

**DOI:** 10.1038/s41598-018-34002-9

**Published:** 2018-10-25

**Authors:** Philipp Baumgartner, Mohamad El Amki, Oliver Bracko, Andreas R. Luft, Susanne Wegener

**Affiliations:** 1 Department of Neurology, University Hospital and University of Zurich, Zurich, 8006 Switzerland; 2 Present Address: Meinig School of Biomedical Engineering, Cornell University, Ithaca, NY14853 United States

## Abstract

Many stroke survivors experience persisting episodic memory disturbances. Since hippocampal and para-hippocampal areas are usually spared from the infarcted area, alterations of memory processing networks remote from the ischemic brain region might be responsible for the observed clinical symptoms. To pinpoint changes in activity of hippocampal connections and their role in post-stroke cognitive impairment, we induced ischemic stroke by occlusion of the middle cerebral artery (MCAO) in adult rats and analyzed the functional and structural consequences using activity-dependent manganese (Mn^2+^) enhanced MRI (MEMRI) along with behavioral and histopathological analysis. MCAO caused stroke lesions of variable extent along with sensorimotor and cognitive deficits. Direct hippocampal injury occurred in some rats, but was no prerequisite for cognitive impairment. In healthy rats, injection of Mn^2+^ into the entorhinal cortex resulted in distribution of the tracer within the hippocampal subfields into the lateral septal nuclei. In MCAO rats, Mn^2+^ accumulated in the ipsilateral thalamus. Histopathological analysis revealed secondary thalamic degeneration 28 days after stroke. Our findings provide *in vivo* evidence that remote sensorimotor stroke modifies the activity of hippocampal-thalamic networks. In addition to potentially reversible alterations in signaling of these connections, structural damage of the thalamus likely reinforces dysfunction of hippocampal-thalamic circuitries.

## Introduction

The majority of stroke patients suffer from transient or permanent post-stroke cognitive impairment, often representing an insurmountable obstacle on the path to independence in daily life or return to work^1–4^. These deficits may be subtle and easily overlooked in the acute phase of stroke, because motor symptoms may be more prominent or because specific cognitive testing is not performed^5,6^. Although higher lesion burden increases the likelihood of cognitive deficits, size or location of the lesion do not reliably predict timing and extent of cognitive impairment after stroke^6–8^. Particularly, the substrates of episodic memory impairment after stroke remain obscure, since the medial temporal lobe (MTL) containing hippocampal and para-hippocampal areas essential for episodic memory formation and retrieval, are usually spared from the ischemic lesion^7,9^. Episodic memory deficits have been detected even in patients with good clinical recovery from stroke and no preexisting cognitive symptoms^10^. In principle, post-stroke memory dysfunction could be explained by (i) direct stroke-induced damage to memory processing brain regions, (ii) secondary decay of memory processing structures remote from but directly structurally connected to the region of primary damage, (iii) neurophysiological alterations in remote regions that are functionally connected to the ischemic area, a phenomenon termed “diaschisis”^11^. Additionally, neuroinflammation occurs in the brain after ischemia, contributing to post-stroke cognitive impairment^12–14^. While direct tissue damage is readily visualized in stroke patients using brain imaging, it is more challenging to detect secondary, more subtle alterations of brain circuitries.

Here, we aimed to characterize hippocampal pathways after sensorimotor stroke by mapping its functional connections using manganese enhanced MRI (MEMRI) in a rat middle cerebral artery (MCA) occlusion model. MEMRI is based on *in vivo* assessment of neuronal circuits and plasticity through the physicochemical properties of manganese (Mn^2+^), a calcium (Ca^2+^) analogue, which enters neurons via Ca^2+^ channels^15,16^. Once inside the cell, it is anterogradely transported along axonal tracts^17^. Based on its paramagnetic properties, Mn^2+^ causes a local reduction in T1 and T2 relaxation times, resulting in signal enhancement on T1-weighted images. Hence, by repeated MR examinations over time, the transport of manganese in neuronal networks can be monitored as an indicator of functional activity^18^.

Our goal was to assess the occurrence of direct hippocampal damage in this stroke model and to reveal remote tissue damage and dysfunction of hippocampal pathways. We here analyzed repeatedly behavioral effects of stroke on sensorimotor and cognitive performance as well as alterations within the MEMRI-labeled functionally interacting hippocampal network for 28 days.

## Materials and Methods

### Animals and stroke induction

All experiments were performed in accordance with the guidelines and regulations approved by the Federal Veterinary Office of Switzerland (Veterinary Office of the Canton of Zurich), animal welfare assurance number ZH 198/2013. Forty-four Sprague-Dawley rats were subjected to either 60 minutes of middle cerebral artery occlusion (MCAO) on the left side (n = 34) or sham surgery (n = 10). A modification of the Koizumi intraluminal filament method was applied^19^. Although laser doppler flowmetry measurements were performed during MCAO, stroke induction was only considered successful if animals showed either a T2 lesion on MRI 27 days after stroke or if a sensorimotor deficit (adhesion tape removal time >15 s or neurological score ≤16 one day after stroke) was observed on day 1 after stroke. Overall, 16 animals had to be excluded, because they either did not survive the perioperative period due to massive stroke or had to be sacrificed because of insufficient weight gain over the 1-month follow-up period (n = 14) or because stroke induction was not successful (n = 2). For final analysis, the MCAO group consisted of 18 animals, while the Sham group consisted of 10 animals.

### Sensorimotor testing

For the sticky tape test, two strips of tape (18 × 12 mm) were applied to both forepaws in random order. The time the animals took to contact (sensory function/neglect) and remove (motor function) the tape on both sides (left and right) was recorded. Before stroke, animals were trained to remove both tapes within 10 seconds (average of three sessions). At each time of testing, we averaged results from three trials, at least 3 minutes apart. The 18-point composite neurological score incorporates the observation of (1) spontaneous activity, (2) symmetry in limb movement, (3) forepaw outstretching, (4) climbing, (5) body proprioception, and (6) response to vibrissae touch. A lower score indicates larger neurological deficits, while a score of 18 points indicates no neurological deficit^20^.

### Novel object recognition test (NORT)

To study recognition memory as a measure of episodic memory in rats, the NORT was performed^21^. One day prior to each test, animals were allowed to explore the arena freely for 30 minutes before returning to their home cages. The following day, the rats were placed in the same arena containing two identical objects placed on each side of the arena, which they were allowed to explore for five minutes. After a 3-hour delay, the animals returned to the arena, with one familiar object exchanged for a novel object in the same position. To offset location or object bias, the novel object and the position (left/right) of the novel object were counterbalanced within each group. Between trials, arena and objects were cleaned using 70% ethanol solution. The test was performed before MCAO or sham operation and again at seven, 14 and 28 days after MCAO/sham, using new pairs of objects for each time point. Recording and scoring: Each trial was recorded with a video camera mounted on the roof above using Noldus EthoVision XT 9.0 video tracking software (Noldus, Wageningen, the Netherlands). Evaluation of exploration time was performed manually with stopwatches. Exploration of an object was defined as sniffing on an object and/or touching it with the  nose. Running around the object, sitting or climbing on it was not considered as exploratory behavior. Velocity of the animals during the trials was automatically recorded by the Noldus EthoVision XT 9.0 video tracking software. For each trial, four animals were placed in four separate cuboid open-field plastic chambers (46 cm–46 cm–40 cm). Each arena was separated from the other arenas by wooden walls (45 cm height), to ensure that no distraction occurred. Both objects were placed on opposite sides on the same position relative to the wall (10 cm × 25 cm away from the wall). Each object was firmly attached to the ground with duct tape. The objects used for the trial consisted of wood, porcelain, class, plastic and metal. They varied in size from 4–5.5 cm in width and 2 cm–5.5 cm in height. The objects were selected on the presumption that each object in the pairs elicits a similar amount of spontaneous investigation.

NORT results were analyzed comparing the amount of time exploring the novel object (T_N_) relative to the total time spent exploring both objects (T_N_ + T_F_) during the test phase. This concept is known as the Recognition Index (RI = T_N_/(T_N_ + T_F_). Animals with RI < 0.6 on day 0 were excluded for NORT analysis.

### Stereotactic manganese injection

Rats were anesthetized using an intraperitoneal administration of a mixture of ketamine (100 mg/kg) and xylazine (13 mg/kg) and were mounted in a stereotaxic frame. Body temperature was maintained at 37 °C using a rectal thermoprobe and a temperature feedback regulator. The skull was exposed, and a burr hole was drilled using a high-speed dental drill at −7.5 mm from bregma, −5.4 mm from sagittal suture to position injections in the entorhinal cortex according to the Paxinos rat brain atlas (Paxinos & Watson, 1997). A 0.5 μL Hamilton syringe was then attached to the stereotactic holder and the needle lowered into the target area (8 mm ventral to the cortical surface). The manganese (MnCl_2_) solution (100 nl of 100 mM) was injected using a microinfusion pump (Harvard PHD 2000). The injection needle was kept in place for two additional minutes before slow withdrawal over 1 min. The correct position of the Manganese injection was then confirmed using MRI and the amount of injected Mn2+ was estimated by measuring signal intensity in the entorhinal cortex directly after Mn2+ injection (Post-Mn).

### Magnetic resonance imaging (MRI)

The first MRI was acquired on day 27 after stroke before MnCl_2_ injection. The follow-up MRIs were performed 30 minutes and 24 hours after manganese injection. MRI was carried out at the biology core laboratory at the University Zurich on a rodent 4.7T MRI-system (Bruker BioSpin GmbH), with a receive-only surface coil. Animals were anesthetized with 2–2.5% isoflurane in a mixture of air and oxygen (fraction of inspired oxygen, 0.25–0.3) and placed in a custom-built cradle. Temperature was maintained at 37 °C using a normothermic blanket (Harvard Apparatus, Edenbridge, Kent, UK). Temperature and respiratory rate were continuously monitored. T2-weighted MRI sequences were obtained using a turbo spin echo sequence with RARE-factor = 4, effective echo time (TE) = 50 ms, repetition time (TR) = 2.9 ms, 5 averages while T1-weighted were obtained using (TE = 2.7 ms, TR = 120 ms). For MEMRI, we used a FLASH 3D sequence, TR 15 ms, TE 2.5 ms, 5 averages, and voxel size of 0.2 × 0.1562 × 0.2083 mm.

### Analysis of T2w weighted MRI (infarct size)

All images were analyzed using ImageJ software (NIH) version 1.50b. T2 lesion size was quantified semi-automatically by delineating the area of T2 hyperintensity with a thresholding tool. Intensity threshold levels were chosen based on visible observation of infarct inclusion and kept constant for each slice and each rat. The area with intensity levels above the chosen threshold was then automatically delineated and summed up by the ImageJ software package. If needed, CSF was manually excluded from the delineated area. Lesion volume in millimeters was then calculated by multiplying total pixel number with pixel size (0.15625 mm × 0.15625 mm) and slice thickness (2 mm).

### Analysis of Manganese enhanced MRI (MEMRI)

To ensure that an adequate amount of Mn^2+^ was injected into the entorhinal cortex, the amount of injected Mn^2+^ was estimated by measuring signal intensity in the entorhinal cortex directly after Mn^2+^ injection (Post-Mn). Two regions of interest (ROIs) per hemisphere were placed onto the entorhinal cortex in the two coronal slices with the maximum signal intensity. The mean intensity of the two ROIs was then calculated. Only rats with an intensity value above a threshold of >11000 A.U. signal intensity over the entorhinal cortex, e.g. sufficient manganese injection, were considered for further analysis. One day later, MRI was repeated and manganese enhancement was evaluated in five coronal and two axial slices. Slices were chosen at different levels from bregma according to the Paxinos and Watson rat atlas (Paxinos and Watson, 1998 and Swanson, 2004). The coronal levels were: bregma and 3.24 mm, 0.24 mm, −2.52 mm, −6.00 mm and −8.04 mm; axial levels were: bregma and 4.8 mm respective 6.3 mm. Matching MRI slices were chosen based on anatomical landmarks and by using a reference slice (bregma −6.24 mm, basilar artery and posterior cerebral arteries were used as anatomical landmarks for identification of the correct slice) and slice thickness (0.1 mm) to ensure correct slice location. MRI slices for ROI analyses were then overlaid onto the Paxinos and Watson brain atlas. In these slices, nine different ROIs were defined. The size of ROIs was defined according to size of the brain structure. Size of the ROIs ranged from 0.15 mm^2^ to 2.40 mm^2^. Some brain regions, such as thalamus and hippocampus, were covered by more than one ROI. The mean intensity of each ROI was then quantified by ImageJ (NIH). Intensity values were normalized in each rat by dividing it by its respective Post-Mn intensity in the entorhinal cortex.

### Tissue staining

After the last MRI scan, rats were anesthetized and perfused with 4% paraformaldehyde in 0.1M PBS. The brains were immersed in ice-cold PBS and transferred to 30% sucrose solution for at least 24 hours. Coronal 40-μm-thick sections were cut on a freezing microtome (Leica, Nussloch, Germany). Slices from seven MCAO animals were of sufficient quality and used for further staining. We carried out immunodetection of mature neurons with the NeuN antibody, mature astrocytes with the GFAP antibody and proliferation marker with the doublecortin (DCX) antibody. Primary antibodies used were mouse NeuN-B clone A60 (1:200, Millipore; MAB377), rabbit GFAP (1:1000, Cell signaling; D1F4Q) and guinea pig anti-Doublecortin (1:1000, Millipore; AB2253). Secondary antibodies were goat α-mouse-Cy3 (1:300, Jackson ImmunoResearch; 115-165-166), goat α-rabbit-Alexa488 (1:300, Jackson ImmunoResearch; 111-225-144) and goat anti guinea pig-Alexa488 (1:300, Abcam; ab150185). Cell nuclei were counterstained with 4,6-diamidino-2-phenylindole (DAPI, Sigma-Aldrich). Images were processed in ImageJ (NIH). For DAB immunohistochemistry, endogenous peroxidase activity was blocked with 2% H_2_O_2_ in phosphate-buffered saline for 60 minutes at room temperature. The regular staining protocol was applied using the biotinylated mouse-anti-rat antibody α-NeuN (1:200, Millipore) as a primary antibody and mouse α-Biotin (1:250; Jackson ImmunoResearch) as a secondary antibody. DAB staining was done using VECTASTAIN Elite ABC Kit (Vector Laboratories, Burlingame, CA, USA) and DAB Peroxidase Substrate Kit, 3,3-diaminobenzidine (Vector Laboratories). Sections were mounted using DPX Mountant (Sigma-Aldrich).

### Immunohistochemical and immunofluorescence analysis

Images were taken using a ×20 objective with a light microscope (Zeiss Imager Z1) for DAB staining and an inverted Leica Wide field microscope (Leica DMI 6000, Leica) for fluorescence staining. ROIs were chosen from hippocampus and thalamus regions bilaterally using the same settings for all conditions. The measurements from ROIs were combined per section and animal. For GFAP and NeuN in the DG, the signal intensity was measured and calculated using the ImageJ (NIH) software package. For the remaining ROIs of NeuN and DCX, cell counts were performed by an experimenter blinded to the experimental groups.

### Experimental Design and Statistical Analysis

**Figure 1:**
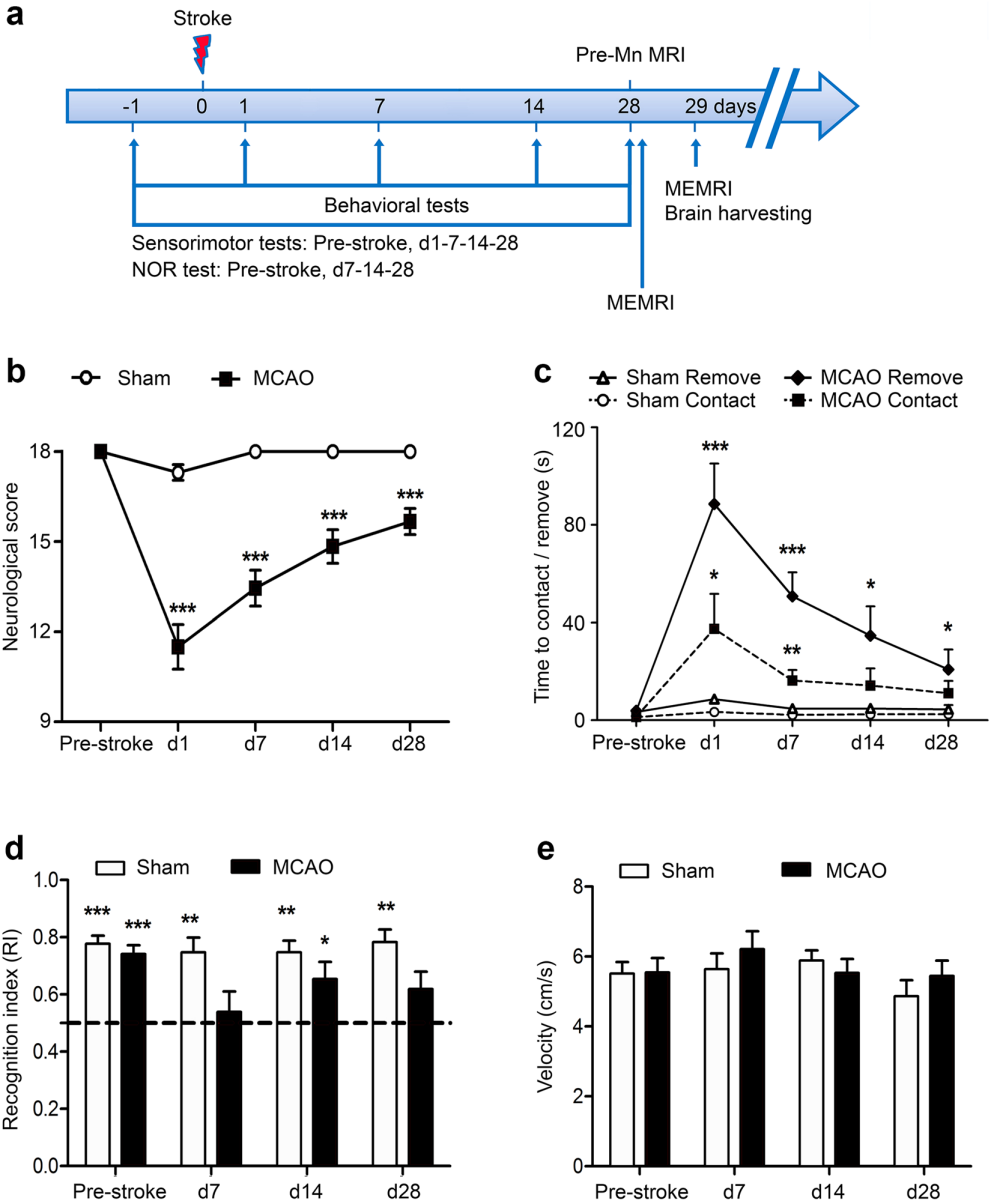
Experimental setup and behavioral test results after MCAO. (**a**) Timeline of experiments. Pre-stroke sensorimotor tests were followed by baseline assessment 1 day before stroke (−1). Following MCAO, behavioral testing was done in all sham (n = 10) and stroke (n = 18) rats on the indicated time points. Pre-Mn MRI = MRI before Manganese injection on day 27 including T1w, T2w, and FLASH 3D FLASH sequences. Injection of MnCl_2_ on day 28 was followed by MEMRI, directly after injection on day 28 as well as on day 29. Animals were sacrificed after the last MRI and brains extracted for further analysis thereafter. (**b**) Results of sensorimotor testing in MCAO (black line, n = 18) versus Sham (white line, n = 10) rats using a composite neurological score where 18 points indicate no deficits and fewer points indicate sensorimotor deficits. (**c**) Time to contact or remove the tape on the impaired forepaw in the sticky tape test (in seconds). (**d**) Cognitive deficits in the NORT showing recognition index (RI) as the time spent actively exploring the new versus the familiar object. Comparison of RI in each group is against chance (RI = 0.5). (**e**) Average velocity of movement during the NORT. MCAO group (n = 12) is resembled by black line, sham group (n = 7) by white line; ***p < 0.001, **p < 0.01, *p < 0.05. Animals with RI < 0.6 on day 0 were excluded for NORT analysis.

The experimental design is shown in Fig. 1a. Rats were first accustomed to the sensorimotor and cognitive function tests. After 60 min MCAO or sham surgery, we assessed behavioral readouts repeatedly for 28 days. On day 27, structural MRI was carried out for infarct volume evaluation as well as pre-Mn^2+^ imaging. MnCl_2_ solution was then injected bilaterally into the entorhinal cortex (EC) and MRI repeated to control for accuracy of the stereotactic injection. One day later, activity-dependent distribution of the Mn^2+^ tracer was visualized using MEMRI and animals were sacrificed thereafter.

Statistical analysis was performed using SPSS Statistics (version 23, IBM, Armon NY, USA). All values are expressed as mean +/− standard error of mean (s.e.m.). For group comparisons, the two-sided independent sample t-test was used. Repeated measures two-way ANOVA was applied to comparisons of behavioral measures over time between groups. The RI from the NORT was compared to chance (0.5) in a one-sample t-test. A p-value < 0.05 was considered significant.

## Results

### MCAO induced sensorimotor and cognitive impairment

MCAO resulted in robust sensorimotor and cognitive deficits (Fig. 1b–d). To make sure that motor dysfunction did not hamper explorative behavior in the novel object recognition test (NORT), velocity of movement in the NORT arena was recorded and shown to be unaffected by MCAO (Fig. 1e).

### Cognitive impairment is not attributable to direct damage of the hippocampal system

**Figure 2:**
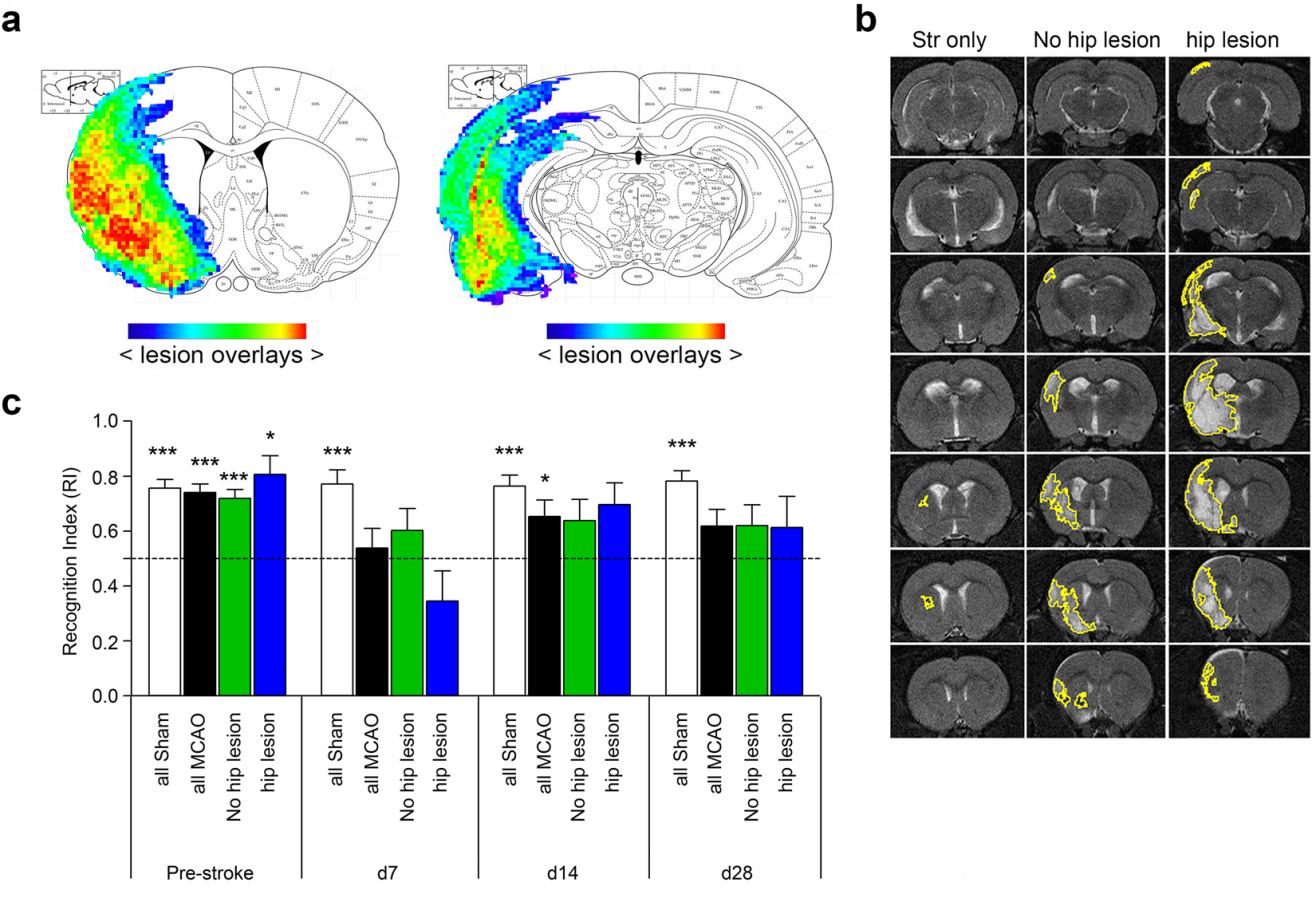
Different stroke patterns and cognitive impairment after MCAO. Stroke patterns were derived from pre-Mn T2-weighted MRI (day 27) after MCAO. (**a**) Two coronal slices from the Paxinos Atlas (Bregma 0.2 mm, Bregma −5.3 mm). Individual lesions from all individual animals are overlaid onto the atlas; with color-coding from blue to red with increasing lesion overlap. (**b**) Representative T2-w images of three animals with different stroke patterns, either involving only striatum (Str), motor cortex and striatum without hippocampal lesion (no hip lesion) or hippocampus (hip lesion), thus indicating additional occlusion of deep cerebral arteries. (**c**) Comparison of NORT performance against chance in animals with different lesion types according to (**b**) at different time points. Error bars represent S.E.M. ***p < 0.001, **p < 0.01, *p < 0.05; n = 12.

First, we analyzed whether cognitive performance in the NORT was associated with direct ischemic damage to the hippocampus. MCAO induces a wide range of infarcts due to differences in vascular anatomy and a model-specific variability of stroke induction^22^, which was explicitly desired in our study (see Fig. 2a,b for overlay of all individual stroke lesions). We observed three characteristic infarct patterns (Fig. 2b): (i) very small lesions restricted to the striatum (n = 3), (ii) striatal and cortical infarcts without involvement of hippocampus (n = 9), and (iii) larger strokes affecting the hippocampus (n = 6). All T2-lesions involved the striatum. In order to analyze if direct affection of the hippocampus was a prerequisite for cognitive impairment, we compared NORT performance between animals with or without hippocampal lesions. We found a reduced mean RI on day 28 in both groups (Fig. 2c). Thus, hippocampal stroke lesion involvement as judged from the T2w MRI did not explain long-term cognitive deficits in NORT.

### MEMRI in the hippocampal system reveals alteration of Mn^2+^ transport after stroke

**Figure 3:**
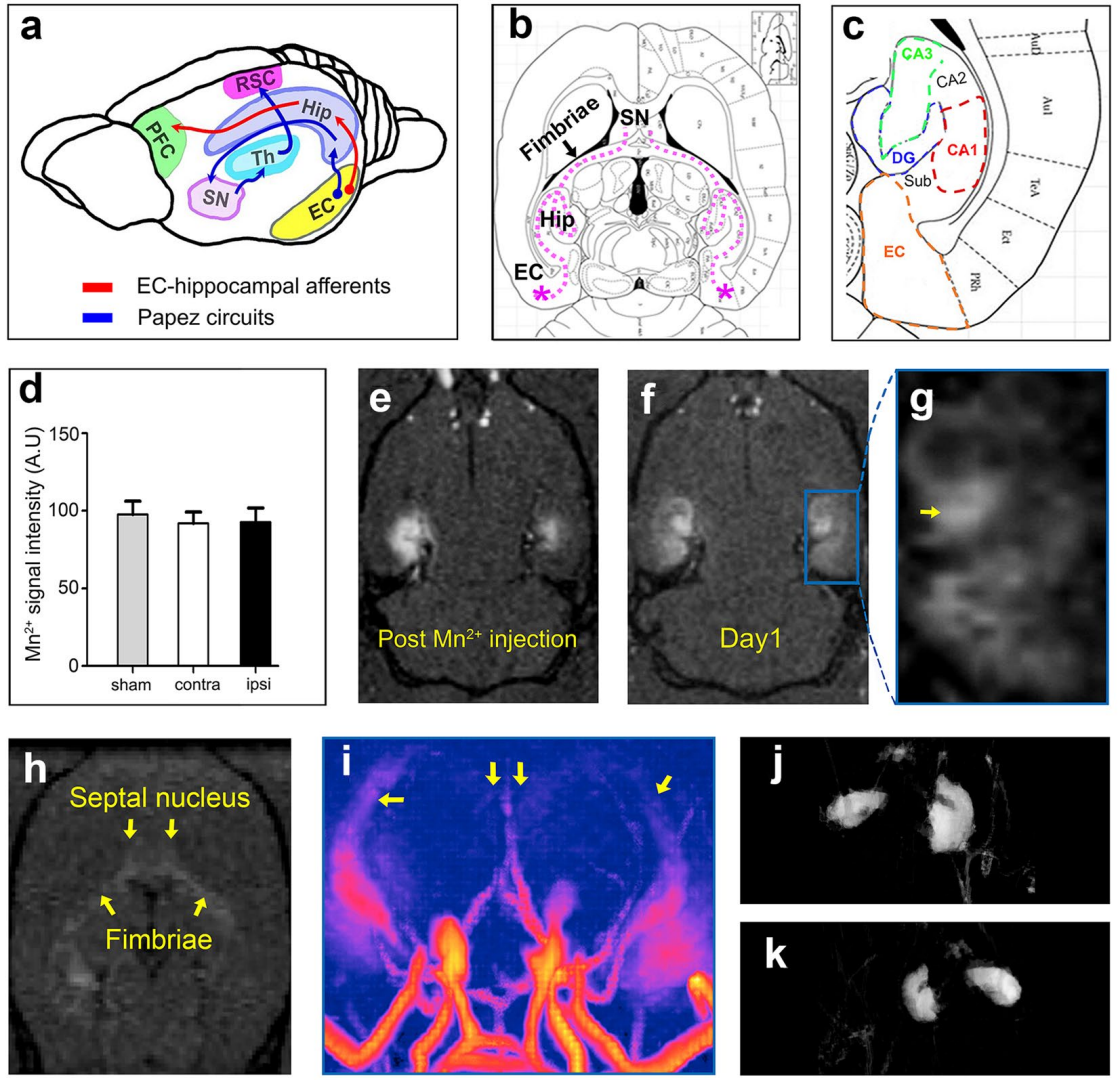
Manganese- enhanced MRI (MEMRI). (**a**) Schematic overview of afferents originating from the entorhinal cortex (EC). SN = lateral septal nuclei, Th = thalamus, Hip = hippocampus, RSC = retrosplenial cortex, PFC = prefrontal cortex. (**b**) Schematic drawing indicating distribution of Mn^2+^-signal after bilateral EC injection. The tracer is transported into the hippocampus (Hip) and then further along the fimbriae hippocampi (Fimbriae) to the SN. (**c**) Enlarged hippocampal anatomy (from Paxinos Rat Brain atlas; Bregma −7.5 mm) indicating hippocampal subfields: DG = Dentate Gyrus; CA1, 2, 3 = respective regions of cornu ammonis, Sub = subiculum. (**d**) Comparison of MEMRI signal intensity after bilateral Mn^2+^ injection into the contralateral versus ipsilateral EC in MCAO rats. The signal is measured in arbitrary units (A.U.). Error bars = S.E.M. (**e**) MEMRI signal directly after injection of 0.1 M MnCl_2_ bilaterally into the EC in a Sham rat. (**f**) Same rat analyzed in MEMRI 24 h later. (**g**) Enlargement of the hippocampus from (**f**). Note the specific distribution of the Mn^2+^ signal throughout the hippocampus and correspondence to anatomical landmarks indicated in (**c**). Yellow arrow points to enhanced DG. (**h**) Same rat as in (**e**,**f**) MEMRI 24 h after bilateral Mn^2+^ injection showing tracer transport to the Fimbriae and SN. (**i**) 3D-reconstruction of the MEMRI signal from the same sham rat as in (**e**–**h**). Yellow arrows point to the enhanced fimbriae and SN. (**j** and **k**) 3D reconstruction of the manganese enhanced parenchyma.

**Figure 4:**
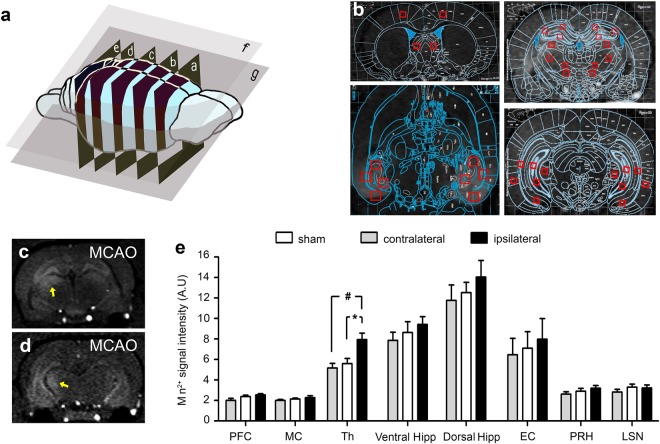
MEMRI signal quantification after MCAO. (**a**) Position of the five axial and two coronal slice positions used for MEMRI-ROI analysis. (**b**) ROI analysis of Mn^2+^-signal on MEMRI. Position of 19 ROIs according to anatomical landmarks. (**c**) MEMRI signal enhancement in the thalamus (arrows) on the ipsilateral side in stroke rats (**c**,**d**). (**e**) MEMRI signal quantification within the different regions of interest in MCAO rats on the ipsilateral (black) versus contralateral (white) side (*p < 0.05) and versus sham operated animals (^#^p < 0.05). The ventral hippocampus consists of ROIs in the CA1, CA3 and DG subregions, the dorsal hippocampus consists of ROIs in the CA1, CA2 and CA3. PFC = Prefrontal cortex; MC = Motor cortex; Th = Thalamus; Vent Hipp = Ventral hippocampus; DG = Dentate Gyrus; CA1, 2, 3 = respective regions of cornu ammonis, Dorsal Hipp = Dorsal hippocampus; EC = Entorhinal cortex; PRH = Perirhinal cortex; LSN = Lateral septal nucleus.

To elucidate alterations in hippocampal network activity, we next performed targeted bilateral injection of MnCl_2_ into the EC and followed the signal distribution using MEMRI. The main components of the afferents projecting from the EC are shown in Fig. 3a. Directly after Mn^2+^ injection, a T1-hyperintense signal was observed within the targeted area of the EC on MR images (Fig. 3e). Quantification of the Mn^2+^ signal intensity within the EC directly after injection showed some inhomogeneity in delivery of the tracer even with constant stereotactic injection procedures. However, on average, the Mn^2+^- induced signal intensity was not different between the ipsi- and contralateral side of stroke as well as compared to SHAM animals (92.59 ± 4.14 A.U. ipsilateral, 91.79 ± 3.70 A.U. contralateral, 97.55 ± 4.90 A.U. sham; Fig. 3d). After one day, the hippocampal formation with its characteristic V-shaped dentate gyrus (DG) together with the band corresponding to the pyramidal cell layer of the cornu ammonis (CA) was clearly enhanced (Fig. 3c,f,g). Mn^2+^ was then further distributed along the fimbriae of the hippocampus to the lateral septal nuclei (Fig. 3b,h,i). For quantification of the MEMRI signal, nine regions of interest (ROI) were placed on both hemispheres according to specified anatomical landmarks (Fig. 4a,b). In contrast to the contralateral, healthy side, we found a significant Mn^2+^ signal enhancement in the ipsilateral thalamus 28 days after stroke (7.93 ± 0.64 A.U. ipsilateral vs. 5.597 ± 0.50 A.U. contralateral, p = 0.0217; Fig. 4c,d,e).

### MCAO causes gliosis in the ipsilesional thalamus

**Figure 5:**
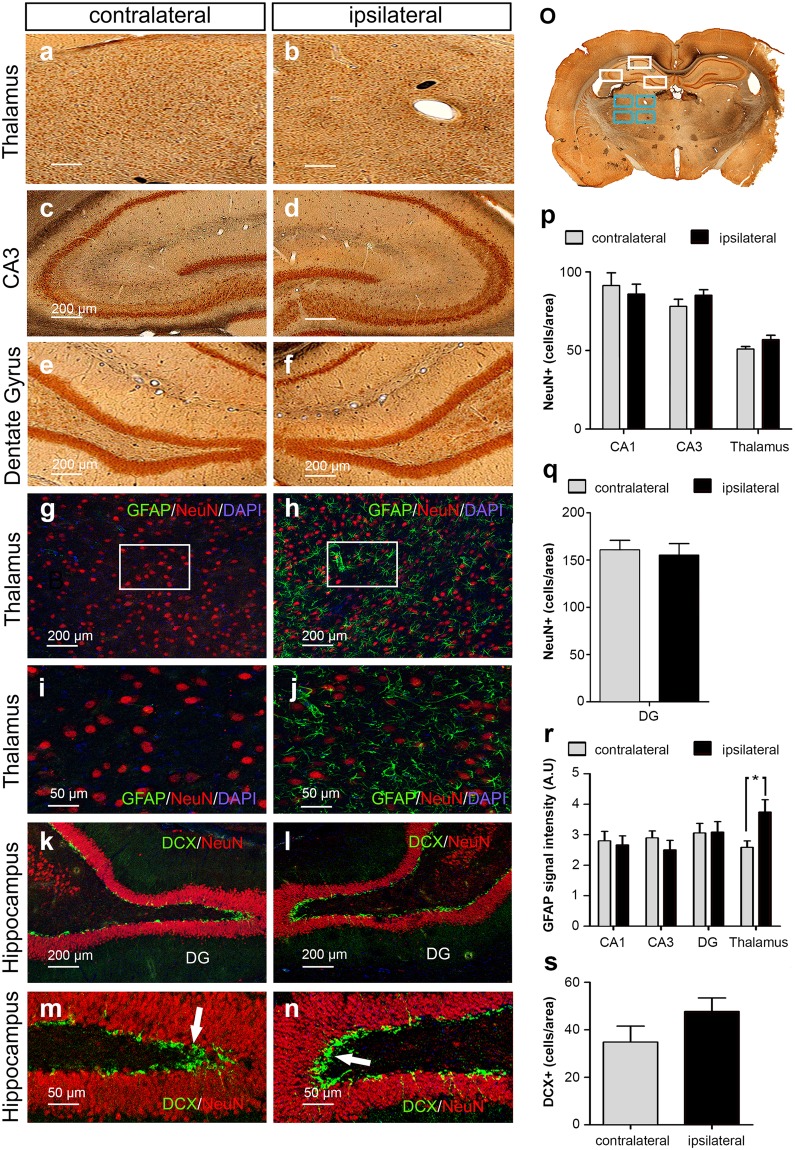
Secondary thalamic degeneration after MCAO. Histologic analysis of brains 4 weeks after MCAO. (**a**–**f**) Representative immunohistochemical images of NeuN expression in the thalamus, CA3 and DG in MCAO animals. (**g**–**j**) Immunofluorescence images demonstrating the expression of NeuN (red), GFAP (green) overlaid onto DAPI on the ipsilesional and contralesional side in the thalamus. Note the increased GFAP signal (green arrows) on the ipsilesional side. (**k**–**n**) Immunofluorescence images demonstrating expression of NeuN (red) and DCX (green) within the ipsilesional and contralesional hippocampus. There is no significant difference in DCX+ cells (white arrows). (**o**) ROI positioning for immunohistochemical/-fluorescence analysis demonstrated on the contralateral side. (**p**) Quantification of NeuN+ cells between the ipsi- and contralesional side in the thalamus, CA3 and CA1 and the NeuN signal intensity in the DG (**q**) in MCAO rats 4 weeks after stroke (n = 7). (**r**) Quantification of GFAP signal intensity in the thalamus and hippocampus. Note the significantly higher GFAP expression in the ipsilesional thalamus. (**s**) Quantification of DCX positive cells between the ipsi- and contralesional hippocampus in MCAO animals (no significant difference). Error bars represent S.E.M. *p < 0.05.

After we had demonstrated that cognitive deficits induced by MCAO are not simply attributable to macroscopic infarct extension into the hippocampal system, we explored structural changes on brain slices four weeks after stroke. First, following MEMRI detection of manganese signal enhancement in the thalamus after stroke, we searched for neuronal death and gliosis within the hippocampus and thalamus. Four ROIs in the thalamus and one ROI in each hippocampal sub-region were analyzed on 3 slices/animal (Fig. 5o). There was no loss of NeuN positive cells in the thalamus (Fig. 5a,b and p) or hippocampus (Fig. 5c–f and p).

However, analysis of GFAP expression revealed considerable gliosis in the ipsilesional thalamus in MCAO animals (Fig. 5g–j). Intensity measurements confirmed that GFAP expression on the ipsilateral side was indeed significantly increased compared to the contralateral side (1.87 ± 0.20 A.U. ipsilateral vs. 1.29 ± 0.11 A.U. contralateral, *p* = 0.0265, Fig. 5r). Strikingly, areas with gliosis corresponded to those areas with accumulation of manganese signal after stroke on MEMRI. There was no difference in GFAP expression within the hippocampal sub-regions (Fig. 5r). To assess the contribution of neurogenesis to differences in cognitive performance, we analyzed DCX expression in the DG on the afflicted hemisphere. DCX positive cells were found abundantly in the DG of each hemisphere (Fig. 5k–n). Counting of DCX positive cells showed no significant differences between the ischemic and contralateral side (Fig. 5s).

## Discussion

**Figure 6:**
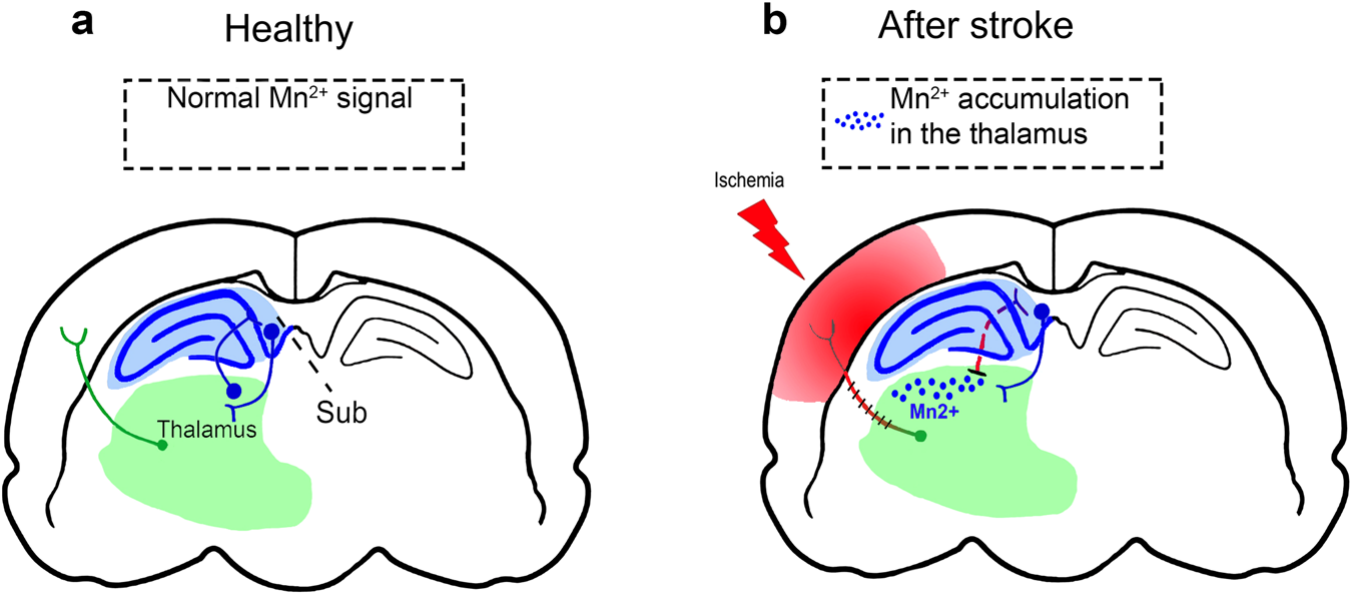
Proposed model of thalamic neurodegeneration effects on MEMRI signal after sensorimotor stroke (**a**) in healthy rats, Mn^2+^ distributes from the EC to the hippocampus, and passes the thalamus via thalamo-subicular (Sub) pathways. (**b**) Ischemic lesions damage reciprocal thalamo-cortical connections. Furthermore, afferent input from the thalamus is reduced due to sensory and motor inactivity. Secondary degeneration contributes to thalamic dysfunction. Therefore, Mn^2+^ (blue) is retained in the thalamus.

In our study, we identified stroke-induced alterations in the activity of hippocampal circuitries. First, we provide further evidence that cognitive deficits imposed by stroke do not simply arise from direct ischemic damage to the hippocampus, but mainly from indirect, delayed distortions of remote, functionally connected regions. MCAO is a variable stroke model, with frequent affection of arteries supplying hippocampal or para-hippocampal areas^22^. However, even without such extensive infarcts and without direct hippocampal damage, cognitive deficits were detected e.g., in purely striatal lesions. Second, using MEMRI, we found a rise of the Mn^2+^ signal in the ipsilesional thalamus of the labeled afferent hippocampal pathways. This could indicate that, while anterograde connections from the hippocampus to the thalamus remain intact, retrograde connections from the thalamus may be malfunctioning after stroke (Fig. 6). Therefore, MCA territory stroke does indeed affect hippocampal-thalamic connections.

Our observations highlight the fact that the hippocampus, a brain structure with decisive role in the formation of memories, does not operate stand-alone, but embedded in a network of extensive and reciprocal connections to the cingulate cortex and the diencephalon^23^. Therefore, injury to the thalamus, through bi-directional tracts to and from the hippocampal subiculum, may cause malfunction of the memory-processing network^24–27^. The structural damage in the ipsilateral thalamus after stroke found in our study goes beyond the classical diaschisis concept, where focal lesions induce remote alterations in neurophysiological function, that recover over time and do not progress into actual tissue breakup^28^. However, transient ipsilateral thalamic hypometabolism has been found in a rat photothrombosis model of stroke without histological signs of tissue damage in this region^29^, indicating that severity, duration or extent of the ischemic damage may determine if remote changes in activity transform into loss of function and cell death. Cell death and structural reorganization in the thalamus after remote stroke have been coined “secondary degeneration”. It appears histologically as astrogliosis, microglial activation and neuronal death^30–33^. Recently, focal thalamic iron accumulation has been visualized on MRI in stroke patients with infarcts initially sparing the thalamus, which was most likely attributable to secondary degeneration as well^34^.

Of note, different neurological conditions may result in similar remote effects: in a model of epilepsy, administration of Mn^2+^ into the EC resulted in a similar pattern of thalamic MEMRI signal accumulation, possibly due to epilepsy-induced reorganization of EC-thalamic pathways^35^. Resting-state EEG recordings in stroke patients have shown that cortical stroke affects thalamic function leading to a condition called thalamo-cortical dysrhythmia, an imbalance between excitation and inhibition of thalamic neurons^36^. This phenomenon can arise due to a lack of excitatory input to the thalamus and has been linked to non-specific symptoms after stroke not attributable the ischemic lesion itself such as chronic pain, mood-related disorders and cognitive impairment. Besides this focal dysregulation after stroke, global conditions such as neuroinflammation, endothelial dysfunction and neurohormonal dysregulation are likely to contribute to post-stroke cognitive impairment^12–14,37,38^.

Could we improve the observed dysfunction of hippocampal-thalamic connections after stroke? A very exciting possibility comes from recent optogenetic experiments targeted at malfunctioning thalamo-cortical fiber tracts after experimental stroke: stimulation of these afferents resulted in synaptic strengthening and improved sensorimotor function of the affected forepaw^39^. Rehabilitation could booster activation of thalamo-cortical inputs and could potentially influence hippocampal-thalamic networks as well. Mn^2+^ enhanced MRI is a well suited tool to demonstrate plasticity of thalamic connections over time along with potential therapeutic interventions^15^.

## Data Availability

The datasets generated during and/or analysed during the current study are available from the corresponding author on reasonable request.
